# Modeling emotion-creativity interaction following brief training

**DOI:** 10.1186/1471-2202-15-S1-P32

**Published:** 2014-07-21

**Authors:** Xiaoqian Ding, Rongxiang Tang, ChangHao Jiang, Yi-Yuan Tang

**Affiliations:** 1 Department of Physics, Dalian University of Technology, Dalian 116024, China; 2 Department of Psychology, University of Texas at Austin, Austin, TX78705, USA; 3 Capital University of Physical Education and Sports, Beijing 100191, China; 4 Department of Psychology, Texas Tech University, Lubbock, TX79409, USA

## 

One form of meditation training, the integrative body-mind training (IBMT) has been shown to improve attention, reduce stress and change self-reports of mood [1]. Here, we examine whether short-term IBMT can improve performance related to creativity and determine the role that mood may play in such improvement using the cross-lagged models.

Forty healthy Chinese undergraduates were randomly assigned to short-term IBMT group or a relaxation training (RT) control group. Mood and creativity performance were assessed by the Positive and Negative Affect Schedule [2] and Torrance Tests of Creative Thinking (TTCT) [3] respectively. ANOVAs revealed a group (IBMT vs. RT) × session (pre-training vs. post-training) interaction effect [F(1, 37) = 14.853; *p* < .01] and a session main effect [F(1, 37) = 36.156; *p* < .01] for TTCT. These results indicated that short-term (30 min per day for 7 days) IBMT improved creativity performance in the divergent thinking task than RT. The ANOVAs also revealed a group (IBMT vs. RT) × session (pre-training vs. post-training) interaction effect and a session main effect for positive affect (PA) and negative affect (NA) (all *p* < .01), indicating better emotional regulation than RT. In addition, the cross-lagged models [4] were used to explore the causal sequence between PA score and TTCT score (Figure 1A) and between NA score and TTCT score (Figure 1B) before and after IBMT training. The synchronous correlations (r PA-before × TTCT-before = .468, r PA-after × TTCT-after = .533; r NA-before × TTCT-before = -.499, r NA-after × TTCT-after = -.633) and the autocorrelations (r PA-before × PA-after = .823, r TTCT-before × TTCT-after = .591; r NA-before × NA-after = .705, r TTCT-before × TTCT-after = .591) were high in magnitude and statistically significant in the non-cross direction, which provides preliminary support for cross-lagged panel correlation.

**Figure 1:**
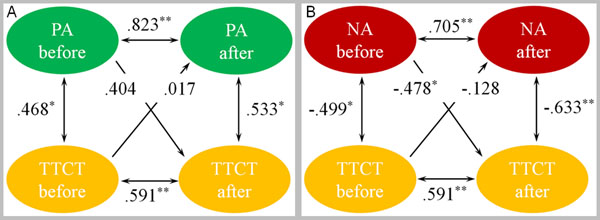
The causal sequence between PA score and TTCT score (A) and between NA score and TTCT score (B) before and after IBMT training. Ellipses indicate measured variables; Arrows depict hypothesized directional or “causal” links/associations; Numbers above or near measured variables represent the correlations or regressions. Spearman’s correlation coefficient and the standardized regression coefficient are used and estimates are statistically significant at *p < .05 and **p < .01.

As predicted, PA had a positive cross-lagged impact on TTCT, which indicated a causal influence from positive mood changes to the creativity changes in IBMT group but not in RT group. In addition, NA had a negative cross-lagged impact on TTCT, which indicated a causal influence from negative mood changes to the creativity changes in the IBMT group but not in RT group.

## Conclusion

Consistent with our previous research, the IBMT group significantly outperformed the RT group in TTCT scores and emotion after training. The cross-lagged analyses indicated that both positive and negative mood changes may contribute to the creativity changes following IBMT. Our results suggested that emotion-related creativity-promoting mechanism may be attributed to short-term meditation. Modeling emotion-creativity interaction using cross-lagged analysis may open up an important avenue for studying meditation-emotion-creativity relationships.
